# Comparison of 12-month therapeutic effect of conbercept and ranibizumab for diabetic macular edema: a real-life clinical practice study

**DOI:** 10.1186/s12886-017-0554-8

**Published:** 2017-08-25

**Authors:** Yule Xu, Ao Rong, Wei Xu, Yunli Niu, Zhen Wang

**Affiliations:** Department of Ophthalmology, Tongji Hospital, Tongji University School of Medicine, 389 Xincun Road, Shanghai, 200065 China

**Keywords:** Conbercept, Diabetic retinopathy, Macular edema, Ranibizumab

## Abstract

**Background:**

To compare the efficacy of intravitreal conbercept and ranibizumab in the treatment of diabetic macular edema (DME) in a real-life clinical practice.

**Methods:**

This was a retrospective study. Among 62 Chinese patients with DME, 32 patients (36 eyes) received intravitreal conbercept (IVC) injections and 30 patients (32 eyes) received intravitreal ranibizumab (IVR) injections, once a month for 3 months followed by as needed therapy. All participants had at least 12 months of follow-up. We compared the changes in best-corrected visual acuity (BCVA) letter score and central retinal thickness (CRT) between groups, as well as the number of intravitreal injections delivered. Safety was assessed with the incidence of adverse events (AEs).

**Results:**

At month 12, the mean BCVA letter score improved by 9.3 ± 5.2 with conbercept, and by 8.9 ± 4.4 with ranibizumab, the mean CRT reduction was 138.4 ± 97.7 μm and 145.2 ± 72.5 μm, respectively. There was no statistically significant difference of improvement in BCVA (*P* = 0.756) and decrease in CRT (*P* = 0.748) between the two groups. The number of intravitreal injections delivered was significantly higher (*P* = 0.027) in the IVR group (7.2 ± 1.0 per eye) than in the IVC group (6.6 ± 0.9 per eye). There were no severe ocular adverse reactions or systemic adverse events.

**Conclusions:**

Both conbercept and ranibizumab are effective in the treatment of DME, achieving the similar clinical efficacy. In comparison to ranibizumab, conbercept shows a longer treatment interval and fewer intravitreal conbercept injections are needed.

**Electronic supplementary material:**

The online version of this article (10.1186/s12886-017-0554-8) contains supplementary material, which is available to authorized users.

## Background

Diabetic macular edema (DME) remains the major cause of visual impairment in patients with diabetic retinopathy [1, 2]. If left untreated, DME may lead to vision loss and blindness, causing significant burdens to the individuals and the society [3]. It arises from the accumulation of plasma constituents and extracellular fluid, as a result of the breakdown of blood-retina barrier [4, 5].

The Early Treatment Diabetic Retinopathy Study (ETDRS) showed the benefit of focal/grid laser for the management of DME, reducing the risk of visual loss [6]. But Laser photocoagulation has limited effects on improving vision. Although corticosteroid is effective in treating DME, benefits are often limited by the common side effects of cataract formation and elevation of intraocular pressure [7].

Vascular endothelial growth factor (VEGF) plays an important role during the process of vascular permeability in DME [8]. Several studies have reported that anti-VEGF agents (ranibizumab, bevacizumab and aflibercept) are efficacious in the treatment of DME [9–14]. Among those anti-VEGF agents, ranibizumab is a fully humanized monoclonal antibody fragment [15]. The RISE and RIDE studies have demonstrated that intravitreal ranibizumab injections were effective in improving visual acuity in patients with DME [10, 11].

Conbercept (Chengdu Kanghong Biotech Co., Ltd., Sichuan, China) is a novel recombinant fusion protein, which is designed as a decoy receptor composed of the second Ig domain of VEGF receptor 1 and the third and fourth Ig domain of VEGF receptor 2 to the constant region (Fc) of human IgG1 [16]. Intravitreal administration of conbercept has been shown to prevent choroidal neovascularization (CNV) growth and leakage in non-human primate [16, 17]. Recently, a series of studies [18–20] have manifested the efficacy and safety of conbercept in treating neovascular age-related macular degeneration (AMD). Chen X et al. also indicates that conbercept can inhibit human retinal endothelial cells (HRECs) migration and sprouting induced by high glucose, through binding VEGF and placental growth factor [21]. These data suggest that conbercept could serve as a new antiangiogenic agent and could be beneficial for diabetic retinopathy. The potential therapeutic effects of conbercept on DME so far have not been assessed.

This study is performed to evaluate the efficacy of conbercept compared with ranibizumab in the treatment of DME.

## Methods

This was a retrospective study performed at the Department of Ophthalmology of the Tongji Hospital Affiliated to Tongji University (Shanghai, China). We reviewed the medical records of all patients with center-involved DME, who received initial injection of conbercept or ranibizumab between 01.05.2014 and 30.09.2015, and had at least 12 months of follow-up. Conbercept and ranibizumab were still ‘off-the-label’ in the treatment of DME in China. This study was conducted according to the ethical standards laid down in the 1964 Declaration of Helsinki and was approved by the Institutional Review Board/Ethics Committee of the Tongji Hospital. After discussion with the patient regarding the potential benefits, risks, off-label use and the alternatives to treatment, informed consent was obtained from each patient.

Patients were aged ≥18 years and diagnosed with either type 1 or 2 diabetes mellitus. The glycosylated hemoglobin (HbA1c) was controlled at ≤10% for at least 3 months before the injection and during the treatment period. Visual impairments were owing to focal/diffuse DME involving the center of the macula and not from the other causes. The diagnosis of DME was based on the characteristic clinical, optical coherence tomography (OCT) and fluorescein angiographic (FA) features. All eyes included in the study should have a central retinal thickness (CRT) ≥ 300 μm measured by OCT and best-corrected visual acuity (BCVA) letter score between 78 and 24 measured by the ETDRS protocol. Exclusion criteria included: previous macular or panretinal laser photocoagulation, history of ocular hypertension or glaucoma, prior intraocular injection of anti-VEGF or steroids, intraocular surgery performed within the last 6 months, presence of high-risk proliferative diabetic retinopathy or significant media opacity, and cataract surgery during the period of follow-up. Patients were also excluded because of insufficient clinical records or intermediate discontinuation of treatment. Finally, 62 patients (68 eyes) with DME were included in this study. Thirty-six eyes of 32 patients were treated with intravitreal conbercept (IVC) and 32 eyes of 30 patients with intravitreal ranibizumab (IVR). All the patients received the same agent they choose during the treatment and were not switched to the other. The choice of agent was determined at the discretion of patients according to their circumstance and wishes.

All eyes included in this study were initiated with 3 monthly intravitreal injections of either 0.5 mg conbercept or 0.5 mg ranibizumab, afterwards treatment was continued in a pro re nata (PRN) regimen. Retreatment criteria was defined as a decrease of visual acuity associated with OCT evidence of increasing CRT (≥ 50 μm) compared with the lowest previous measurement.

At baseline and each month visit during the follow-up, patients received a complete ophthalmologic examination, including slit-lamp biomicroscopy, BCVA, dilated funduscopic examination, OCT, and fundus photography. In addition, FA was performed at the discretion of the researcher and not at each month visit. BCVA was assessed using the ETDRS visual acuity chart at 4 m. CRT was measured by Cirrus HD-OCT (Carl Zeiss, Meditec, Dublin, CA), with software version 4.0. Retinal thickness of the central 1 mm diameter area was obtained for analysis.

The intravitreal injection procedure was performed under aseptic conditions in an operating room, which include the use of sterile gloves and a sterile drape. After adequate topical anesthesia and eyelid speculum insertion, a single dose of 0.5 mg (0.05 ml) conbercept or ranibizumab was injected intravitreally 3.5 to 4 mm posterior to the limbus with a 30-gauge needle. All eyes underwent an ocular examination at one and seven days after each injection for intraocular pressure rise and anterior chamber reaction. All ocular and systemic adverse events (AEs), including information on their relationship to the agents and procedure, were recorded at every visit.

Statistical analysis was performed by SPSS software, version 17.0 (SPSS, Inc., Chicago, IL, USA). Differences in categorical variables were assessed with the chi-square test. The paired samples t-test was used to compare the BCVA and the CRT to baseline values in each treatment group. The independent samples t-test was performed to determine statistically significant differences between two groups as regards mean change in BCVA and CRT. All statistical tests were 2-sided. A *P* value of less than 0.05 was considered to indicate statistical significance.

## Results

Sixty-eight eyes of 62 patients treated with conbercept or ranibizumab were included in this study. All participants had at least 12 months of follow-up. The mean age of the patients was 61.0 ± 13.2 years; 45.2% were women, and 54.8% were men. At baseline, 57 of the patients (91.9%) had type 2 diabetes and 5 of the patients (8.1%) had type 1 diabetes. The mean duration of diabetes was 13.8 ± 5.5 years. The mean BCVA letter score was 48.1 ± 10.6 and the mean CRT was 471.5 ± 103.7 μm. The baseline characteristics of the DME patients were summarized in Table 1. The two treatment groups were well balanced for demographics and ocular characteristics.

**Table 1:** Baseline Characteristics of Patients with DME Included in Two Treatment Groups

Characteristic	IVC Group	IVR Group	*P* value
Number of patients, n	32	30	—
Number of eyes, n	36	32	—
Mean age ± SD (years)	61.3 ± 14.9	60.7 ± 11.4	0.880*
Gender, n (%)			
Men	18(56.3)	16(53.3)	
Women	14(43.7)	14(46.7)	0.818**
Diabetes type, n (%)			
Type I	3(9.4)	2(6.7)	
Type II	29(90.6)	28(93.3)	—
DME type, n (%)			
Focal	12(33.3)	9(28.1)	
Diffuse	24(66.7)	23(71.9)	0.643**
mean HbA1c ± SD	7.9 ± 1.0	7.8 ± 0.8	0.904*
Mean duration of diabetes ± SD (years)	14.4 ± 6.8	13.2 ± 3.6	0.372*
Mean duration of DME ± SD (months)	4.2 ± 3.7	4.4 ± 3.0	0.847*
Mean BCVA ± SD (letter score)	49.4 ± 10.3	46.6 ± 10.9	0.272*
Mean CRT ± SD (μm)	469.3 ± 107.7	473.9 ± 100.6	0.858*

*DME* diabetic macular edema, *IVC* intravitreal conbercept, *IVR* intravitreal ranibizumab, *SD* standard deviation, *HbA1c* hemoglobinA1c, *BCVA* best-corrected visual acuity, *CRT* central retinal thickness

*Independent t test; **chi-square analysis

**Fig. 1:**
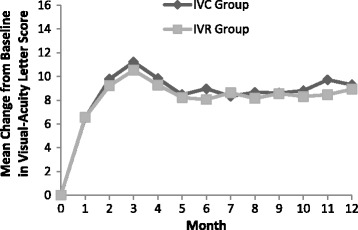
Mean change in best-corrected visual acuity (BCVA). IVC = intravitreal conbercept; IVR = intravitreal ranibizumab

The average level of BCVA letter score improvement over all monthly post-baseline assessments from month 1 to month 12 was shown in Fig. 1. One month after treatment, statistically significant (*p* < 0.001) improvements in the BCVA letter score were observed for both the IVC group (6.5 ± 3.3) and the IVR group (6.6 ± 2.8). These improvements were continued up to month 3 and were sustained until the last assessment time point at month 12 (Fig. 1). At month 12, the improvement in the BCVA letter score was 9.3 ± 5.2 in the IVC group (*P* < 0.001) and 8.9 ± 4.4 in the IVR group (*P* < 0.001). There was no statistically significant difference of improvement in BCVA between two groups (*P* = 0.756, Table 2). At the time of month 12 visit, 30 eyes (83.3%) gained ≥5 ETDRS letters, 15 eyes (41.7%) gained ≥10 ETDRS letters, 7 eyes (19.4%) gained ≥15 ETDRS letters in the IVC group, and 26 eyes (81.3%) gained ≥5 ETDRS letters, 13 eyes (40.6%) gained ≥10 ETDRS letters, 5 eyes (15.6%) gained ≥15 ETDRS letters in the IVR group (Table 2).

**Table 2:** Best-Corrected Visual Acuity and Central Retinal Thickness Outcome at Month 12

Characteristic	IVC Group (*n* = 36)	IVR Group (*n* = 32)
Mean BCVA letter score at month 12 ± SD	58.7 ± 8.0	55.5 ± 9.6
Mean CRT at month 12 ± SD, μm	330.9 ± 77.2	328.7 ± 71.9
Mean change in BCVA letter score from baseline to month 12		
Mean ± SD	9.3 ± 5.2	8.9 ± 4.4
*P* value	0.756^a^	
Mean CRT change from baseline to month 12 ± SD, μm		
Mean ± SD	−138.4 ± 97.7	−145.2 ± 72.5
*P* value	0.748^a^	
Categorized BCVA letter score outcome at month 12, n (%)		
Gain of ≥5	30 (83.3)	26 (81.3)
Gain of ≥10	15 (41.7)	13 (40.6)
Gain of ≥15	7 (19.4)	5 (15.6)

*IVC* intravitreal conbercept, *IVR* intravitreal ranibizumab, *BCVA* best-corrected visual acuity, *CRT* central retinal thickness, *SD* standard deviation

^a^Independent t test

**Fig. 2:**
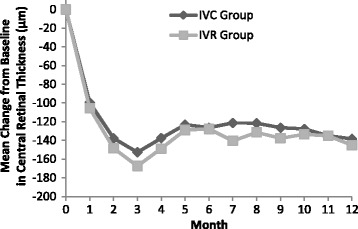
Mean change in central retinal thickness (CRT). IVC = intravitreal conbercept; IVR = intravitreal ranibizumab

The mean change in CRT over 12 months of follow-up was shown in Fig. 2. At month 12, the mean CRT reduction was 138.4 ± 97.7 μm in the IVC group and 145.2 ± 72.5 μm in the IVR group. There was no significant difference of decrease in CRT between two groups (*P* = 0.748, Table 2).

In our study, the mean number of injections delivered was significantly higher (*P* = 0.027) in the IVR group (7.2 ± 1.0 per eye) than in the IVC group (6.6 ± 0.9 per eye). In the IVC group, 6 eyes (16.7%) received 8 injections, 15 eyes (41.7%) received 7 injections, 11 eyes (30.5%) received 6 injections and 4 eyes (11.1%) received 5 injections over the 12 months study period. In the IVR group, 2 eyes (6.3%) received 9 injections, 10 eyes (31.2%) received 8 injections, 13 eyes (40.6%) received 7 injections, 5 eyes (15.6%) received 6 injections and 2 eyes (6.3%) received 5 injections.

Major ocular and systemic AEs were summarized in Table 3. Seventeen patients (53.1%) in the IVC group and 14 patients (46.7%) in the IVR group experienced at least 1 AE. There were no reports of serious complications related to intravitreal injections during the 12 months of follow-up, such as rhegmatogenous detachment or endophthalmitis. Eye pain, intraocular pressure increase and conjunctival hemorrhage were the common ocular adverse events in both groups. Mild vitreous hemorrhage was observed in 1 eye in the IVC group. Systemic AEs were infrequent in both treatment groups (Table 3). Two patients in the IVC group and 3 patients in the IVR group were reported as having hypertension. There were no cases of arterial thromboembolic events in any of the treatment group.

**Table 3:** Ocular and Systemic Adverse Events through 12 Months

Event	IVC Group (*n* = 32)	IVR Group (*n* = 30)
Patients with at least 1 AE	17 (53.1%)	14 (46.7%)
Prespecified ocular adverse events		
Intraocular pressure increased	4	3
Eye pain	5	4
Conjunctival hemorrhage	5	3
Vitreous hemorrhage	1	0
Vitreous floaters	1	1
Vitreous detachment	0	1
Macular fibrosis	1	0
Corneal abrasion	1	1
Dry eye	1	0
Systemic events		
Hypertension	2	3
Nasopharyngitis	2	1
Bronchitis	0	1
Headache	1	1
Influenza	1	2
Back pain	0	1
Pneumonia	1	0
Hypoglycemia	1	1

*AE* adverse event, *IVC* intravitreal conbercept, *IVR* intravitreal ranibizumab

## Discussion

VEGF is considered a highly specific vascular endothelial cellular regulatory factor that is closely related to angiogenesis and vascular permeability in diabetic retinopathy. VEGF family members, especially VEGF-A isoforms, are the principal stimulators of pathological angiogenesis [22]. Placental growth factor is another member of the VEGF family, which play an important role in ocular neovascularization and vascular permeability [23]. Ranibizumab is a recombinant humanized monoclonal antibody fragment with high binding affinity for human VEGF-A. Conbercept is designed as a receptor decoy that aims and binds to all VEGF-A isoforms, VEGF-B, as well as placental growth factor. Compared with ranibizumab (48 kDa), conbercept (143 kDa) is larger. Its affinity for VEGF is 30-times that of ranibizumab [16]. Theoretically, this leads to a more sustained VEGF inhibition. Although conbercept has been approved to treat neovascular AMD, similar studies concerning conbercept in the treatment of DME are lacking. In this study, we compared the efficacy of conbercept with that of ranibizumab in patients with DME. The improvement in visual acuity from baseline to month 12 was 9.3 letters, and 8.9 letters respectively. This result proved the effectiveness of intravitreal conbercept for the treatment of DME. Moreover, conbercept appeared to have similar visual and anatomic outcomes with ranibizumab.

The efficacy results of conbercept/ranibizumab treatment from this research are similar with several recently published studies. In the REVEAL study, which ranibizumab (0.5 mg) was administered with 3 monthly injections followed by individualized PRN therapy in Asian patients with DME, the mean change in visual acuity from baseline to month 12 was 6.6 letters [24]. In the RESOLVE study, where 0.5 mg ranibizumab was given for 3 months then PRN in non-Asian patients, the mean change in visual acuity from baseline to month 12 was 6.8 letters [25]. In the DRCR-T study, which ranibizumab (0.3 mg) was administered as frequent as every 4 weeks, the mean improvement in the visual acuity at one year was 11.2 letters [14]. Similar to conbercept, aflibercept is also designed as a receptor decoy [26]. In the DA VINCI Study, the mean increase in visual acuity over 1 year was 12.0 letters in patients with DME who received 2 mg aflibercept for 3 months and then on PRN basis [27]. The differences in the visual acuity outcome among those studies may be partly due to the differences in study designs, baseline characteristics, inclusion and exclusion criteria, and treatment regimens.

The vitreous half-life of ranibizumab is 2.88 days in rabbits [28], shorter than that of conbercept (4.2 days) [29]. Zhang M et al. also indicates that a single intravitreal conbercept injection (0.5 mg) in monkey may have an inhibitory effect against VEGF over 81 days [17]. In the REVEAL study, an average of 7.8 intravitreal injections was needed in the ranibizumab treatment arm over 12 months [24]. In the RESOLVE study, a mean of 7.0 intravitreal injections was delivered in the ranibizumab arm [25]. In our study, both conbercept and ranibizumab were initiated with 3 monthly injections in a fixed loading phase followed by as needed therapy. We found that the number of intravitreal injections delivered was significantly higher in the IVR group (7.2 ± 1.0) than in the IVC group (6.6 ± 0.9) throughout the 12-month study period. Conbercept showed a longer treatment interval between dosing. In the PHOENIX study for treating neovascular AMD, participants responded well to conbercept even with 3-month intervals following a fixed loading phase of 3 monthly injections [20]. Longer intervals between dosing may provide advantages compared with monthly dosing in terms of a decreased number of injections, which not only reduces the possible adverse effects of intravitreal injections, but also minimizes the financial cost. In addition, Longer-term effects of conbercept mean less clinic visit.

Both conbercept and ranibizumab were safe and well tolerated in our study. The ocular AEs were typical of those associated with intravitreal injections such as intraocular pressure increase, eye pain, conjunctival hemorrhage, etc. There were no reports of rhegmatogenous detachment or endophthalmitis. Systemic AEs were infrequent in both groups. As the true incidence of ocular and systemic AEs requires a large-scale trial for accurate assessment, the adverse events might be limited by the small number of patients in this study.

The limitations of the study are the nonrandomized retrospective design and the short-term follow-up, which preclude any estimate of the safety and efficacy of conbercept for DME. Moreover, the investigators were not masked to treatment modality. A large head-to-head study between conbercept and ranibizumab in patients with DME would be ideal to verify and confirm the results of the present study.

## Conclusions

This preliminary study indicates that both conbercept and ranibizumab are effective in the treatment of DME, achieving the similar clinical efficacy. In comparison to ranibizumab, conbercept shows a longer treatment interval and fewer intravitreal conbercept injections are needed in clinical practice.

## Additional files

Additional file 1: Table S1. Main outcome measures of 12 month follow-up. Excel table including the raw data of the changes in best-corrected visual acuity letter score and central retinal thickness, as well as the number of intravitreal injections delivered. (XLSX 20 kb)

## Abbreviations

AEs: Adverse events
AMD: Age-related macular degeneration
BCVA: Best-corrected visual acuity
CNV: Choroidal neovascularization
CRT: Central retinal thickness
DME: Diabetic macular edema
ETDRS: Early treatment diabetic retinopathy study
FA: Fluorescein angiographic
HRECs: Human retinal endothelial cells
IVC: Intravitreal conbercept
IVR: Intravitreal ranibizumab
OCT: Optical coherence tomography
PRN: pro re nata
VEGF: Vascular endothelial growth factor
